# Multimodal imaging findings of intrahepatic cholangiocarcinoma arising from a biliary adenofibroma: a case report with radiological–pathological correlation

**DOI:** 10.1007/s00261-023-03908-y

**Published:** 2023-04-19

**Authors:** Noriko Kanemaru, Yudai Nakai, Takeyuki Watadani, Takahiro Nakao, Munetoshi Hinata, Akiko Nakazawa, Nobuhisa Akamatsu, Tetsuo Ushiku, Kiyoshi Hasegawa, Osamu Abe

**Affiliations:** 1 Department of Radiology, Graduate School of Medicine, The University of Tokyo, 7-3-1 Hongo, Bunkyo-ku, Tokyo, 113-8655 Japan; 2 Department of Computational Diagnostic Radiology and Preventive Medicine, The University of Tokyo Hospital, 7-3-1 Hongo, Bunkyo-ku, Tokyo, 113-8655 Japan; 3 Hepato-Biliary-Pancreatic Surgery Division, Department of Surgery, Graduate School of Medicine, The University of Tokyo, 7-3-1 Hongo, Bunkyo-ku, Tokyo, 113-8655 Japan; 4 Department of Pathology, Graduate School of Medicine, The University of Tokyo, 7-3-1 Hongo, Bunkyo-ku, Tokyo, 113-8655 Japan

**Keywords:** Biliary adenofibroma, Malignant transformation, Computed tomography, Magnetic resonance imaging, Fluorine-18-2-deoxy-d-glucose positron emission tomography and computed tomography

## Abstract

**Purpose:**

Biliary adenofibroma is a solid microcystic epithelial neoplasm in the liver, comprising microcystic and tubuloacinar glandular tissues lined by a non-mucin secreting biliary epithelium and supported by a fibrous stroma. It is an extremely rare benign tumor with potential for malignant transformation. Herein, we report the case of a 64-year-old woman diagnosed with intrahepatic cholangiocarcinoma arising from biliary adenofibroma.

**Methods:**

Imaging studies revealed a tumor of 50 mm diameter, consisting of two components in S1 of the liver. The ventral portion of the tumor showed an ill-defined mass with early peripheral and gradual centripetal enhancement invading to the middle hepatic vein on computed tomography (CT), diffusion restriction on magnetic resonance images, and high fluorine-18-2-deoxy-d-glucose (FDG) uptake on positron emission tomography, like conventional intrahepatic cholangiocarcinoma. The dorsal portion showed a well-defined and low-attenuated mass with heterogeneous early enhancement and partial wash-out on CT, marked hyperintensity on heavily T2-weighted images, and low FDG uptake. The patient subsequently underwent extended left hepatectomy.

**Results:**

Pathologically, the former was diagnosed as cholangiocarcinoma and the latter as biliary adenofibroma. We discuss the radiological-pathological correlation of the tumor with a literature review.

**Conclusion:**

Preoperative diagnosis of biliary adenofibroma is extremely challenging; however, clinically, it is crucial not to miss the presence of malignant findings.

**Graphical abstract:**

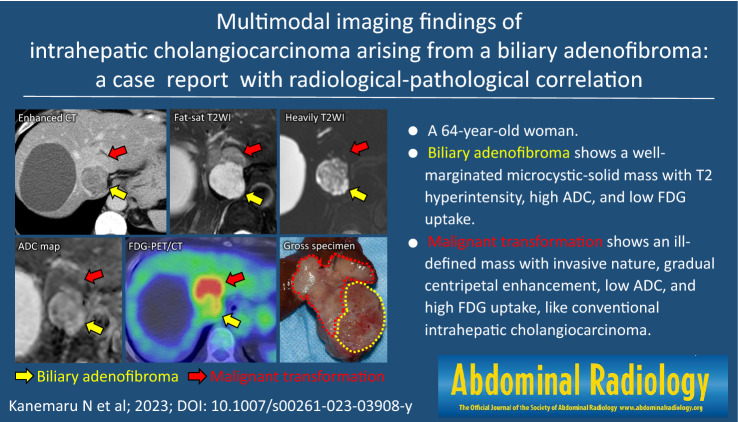

## Introduction

Biliary adenofibroma is a rare, primary hepatic tumor characterized by microcystic and tubuloacinar glandular structures lined by non-mucin-secreting biliary epithelium and supported by a fibroblastic stromal scaffolding [1]. Although usually benign, it has potential for malignant transformation, which makes it important to detect its malignant features. Till date, the radiologic finding of biliary adenofibroma has been reported to be a cystic mass with a well-defined margin, lobulated shape, and internal septa with/without a solid component. In addition, some malignant features have been suggested, which include multiple lesions, a solid component with restricted diffusion on magnetic resonance (MR) images, an obscure margin, a peripheral edematous halo, pseudocapsule formation, and wash-in during the arterial phase followed by wash-out in the venous phase [2]. However, because of its rarity, detailed imaging characteristics of biliary adenofibroma and its malignant component have not been fully clarified. Indeed, for the malignant component, there were few reports with images available (2 cases with ultrasound [3, 4], 8 cases with CT [2–9], 5 cases with MRI [3, 4, 10, 11]). Here, we present the case of a 64-year-old woman who underwent surgery for a hepatic mass that was pathologically diagnosed as cholangiocarcinoma arising from biliary adenofibroma. Moreover, we discuss the imaging features of biliary adenofibroma and its malignant transformation focusing on the radiological-pathological correlation. To the best of our knowledge, this is the first case report with fluorine-18-2-deoxy-d-glucose positron emission tomography and computed tomography (FDG-PET/CT) findings, which might add new insights to the radiological features of biliary adenofibroma with a malignant component.

## Case report

A tumor in S1 of the liver was found on abdominal ultrasound during health examination in a 64-year-old Japanese woman. She did not have any symptoms or notable family, smoking, or drinking history. She had a medical history of appendectomy for acute appendicitis, hysterectomy for uterine myoma, right ovariectomy for ovarian thecoma, right adrenalectomy for Cushing syndrome, and laparoscopic cholecystectomy for a gallbladder polyp. Contrast-enhanced CT performed 9 years ago at the referring hospital did not show any S1 lesions (image not shown). She also had hypertension, hyperlipidemia, and osteoporosis. She had no abnormal physical findings, including lymphadenopathy and hepatosplenomegaly.

Laboratory blood examination revealed that liver enzymes and tumor markers such as alpha-fetoprotein, carbohydrate antigen 19-9, carcinoembryonic antigen, and protein induced by vitamin K absence or antagonist II were within normal limits. She was not infected with hepatitis B and C virus.

**Fig. 1:**
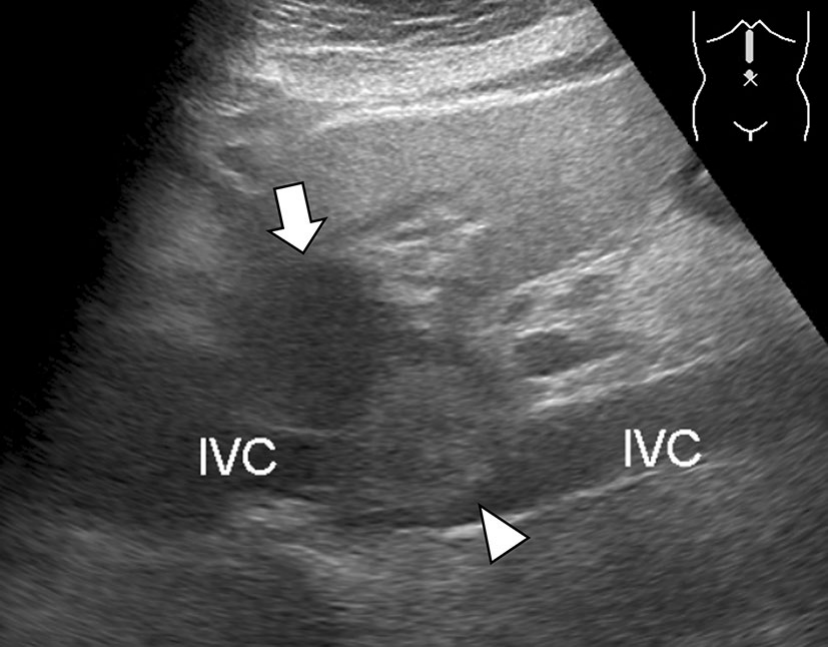
Abdominal ultrasound reveals that the tumor is composed of two distinct components on the ventral and dorsal sides in S1 of liver. The ventral portion shows low echogenicity (arrow) and the dorsal portion shows high echogenicity (arrowhead). The latter component touched widely with the inferior vena cava (IVC), and their boundary is clear

Abdominal ultrasound revealed that the tumor comprised two distinct components on the ventral and dorsal sides in S1 of liver (Fig. 1). The ventral portion showed low echogenicity, and the dorsal portion showed high echogenicity. While the latter component touched widely with the inferior vena cava, their boundary was clear.

**Fig. 2:**
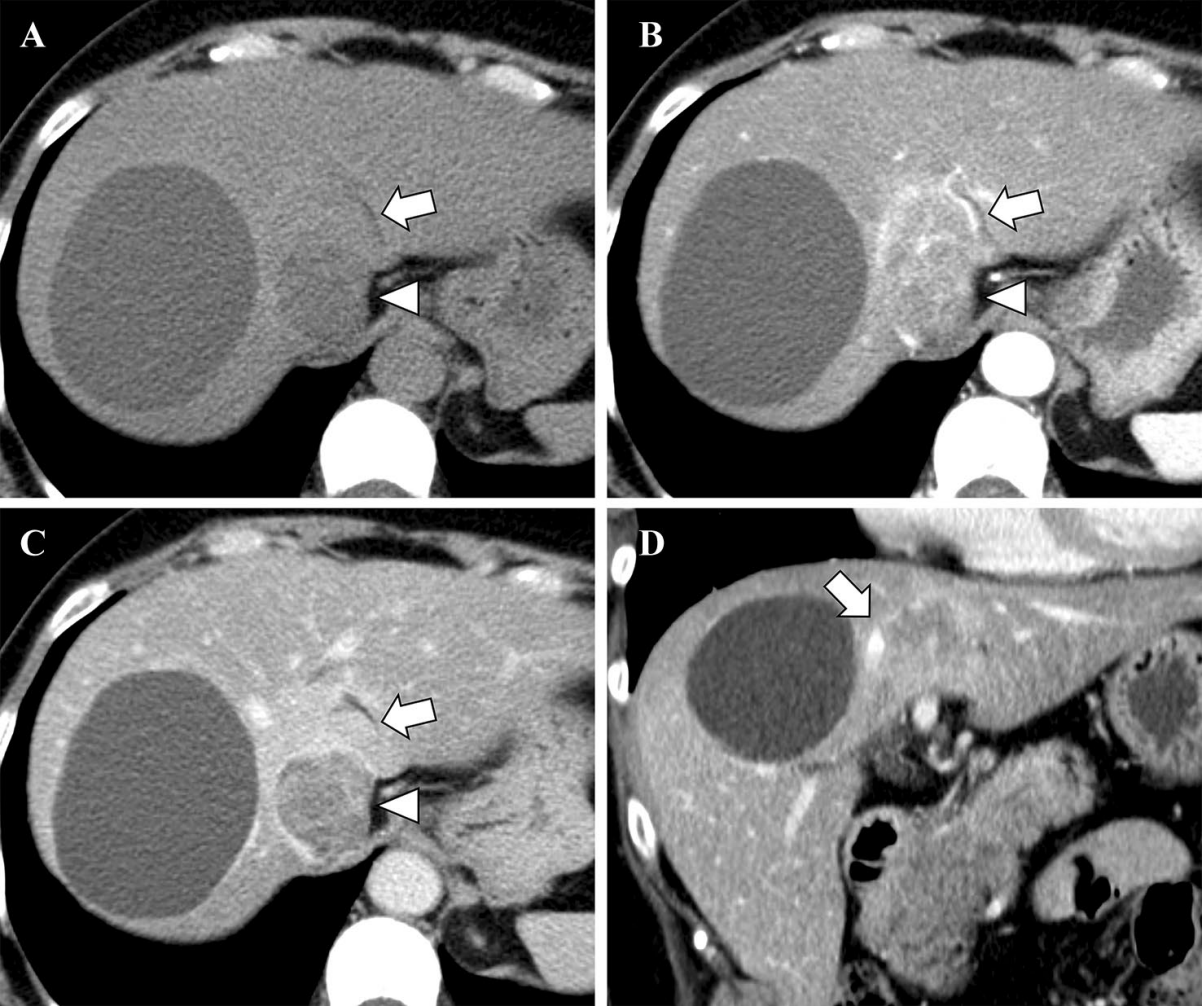
Abdominal CT demonstrates a tumor which is 50 mm in diameter in S1 of the liver. The ventral portion has an irregular margin and shows peripheral early and gradual centripetal enhancement (**A** plain CT; **B** arterial phase; **C** late/equilibrium phase, arrow). In the superior aspect of the lesion, the middle hepatic vein is obstructed, suggesting venous invasion of the tumor (**D** coronal plane in portal phase, arrow). The dorsal portion is a well-defined and low-attenuated mass and shows heterogeneous early enhancement and partial wash-out (**A**–**C** arrowheads)

Abdominal dynamic contrast-enhanced CT was performed with bolus-tracking technique. Following the plain CT, the arterial phase was obtained 15 s after CT value of the abdominal aorta reached the trigger, the portal phase was obtained 40 s after the arterial phase, and the late/equilibrium phase was obtained 180 s after the injection. Abdominal contrast-enhanced CT demonstrated a tumor of 50 mm diameter in S1 of the liver (Fig. 2). The ventral portion had an irregular margin and showed early enhancement predominantly in peripheral area and gradual enhancement in central area. In the superior aspect of the lesion, the middle and left hepatic veins were obstructed and narrowed, respectively, suggesting venous invasion of the tumor. The dorsal portion was a well-defined and low-attenuated mass. It showed heterogeneous early enhancement and partial wash-out.

**Fig. 3:**
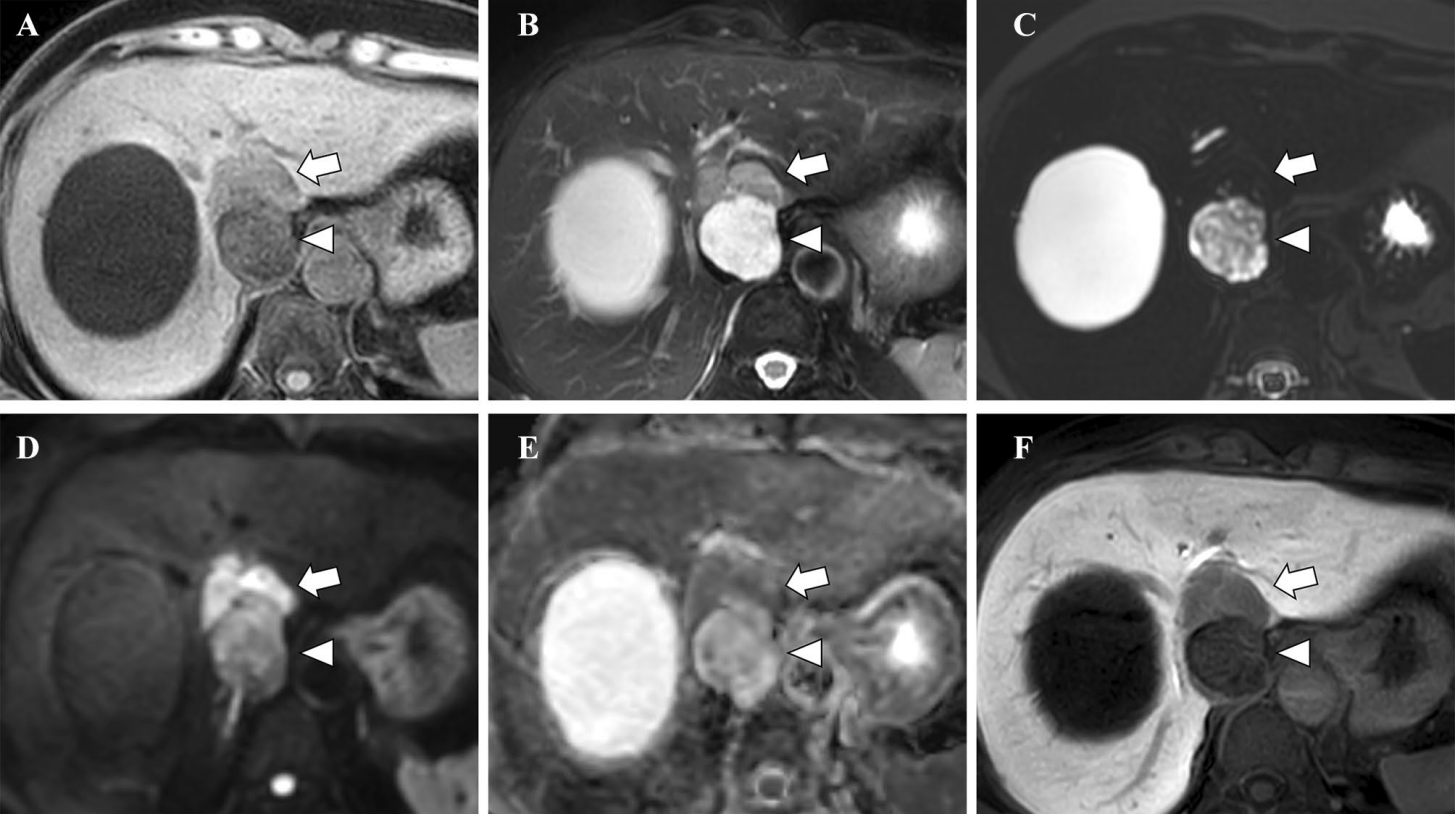
On gadoxetic acid-enhanced MR imaging, the ventral portion exhibited slightly low signal intensity on T1-weighted image (**A** arrow), slightly high signal intensity on fat-saturated T2-weighted image (**B** arrow), markedly low signal intensity on heavily T2-weighted image (**C** arrow), diffusion restriction (**D** diffusion-weighted image [b = 800]; **E** apparent diffusion coefficient [ADC] map, arrow, ADC = 1.1 × 10^−3^ mm^2^/s), and no uptake of gadoxetic acid in the hepatobiliary phase (**F** arrow). The dorsal portion showed low signal intensity on T1-weighted image (**A** arrowhead), high signal intensity on fat-saturated T2-weighted (**B** arrowhead) and heavily T2-weighted image (**B** arrowhead), no diffusion restriction (**D** diffusion-weighted image [b = 800]; **E** ADC map, red arrow, ADC = 1.5–2.5 × 10^−3^ mm^2^/s), and no uptake of gadoxetic acid in the hepatobiliary phase (**F** arrowhead)

On MR imaging, the ventral portion exhibited slightly low signal intensity on T1-weighted images, slightly high signal intensity on T2-weighted images, markedly low signal intensity on heavily T2-weighted images, diffusion restriction (apparent diffusion coefficient [ADC] = 1.1 × 10^−3^ mm^2^/s), and no uptake of gadoxetic acid in the hepatobiliary phase (Fig. 3). The dorsal portion showed low signal intensity on T1-weighted images, high signal intensity on T2-weighted and heavily T2-weighted images, no diffusion restriction (ADC = 1.5–2.5 × 10^−3^ mm^2^/s), and no uptake of gadoxetic acid in the hepatobiliary phase.

**Fig. 4:**
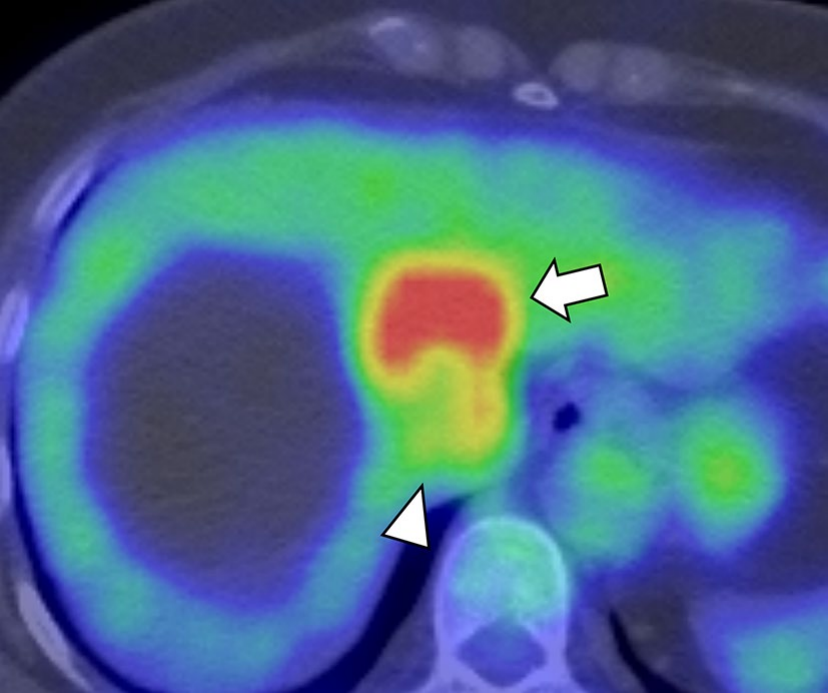
FDG-PET/CT shows high FDG accumulation in the ventral portion (SUV_max_ = 8.3, arrow) and moderate accumulation in the dorsal portion (SUV_max_ = 2.8, arrowhead)

On FDG-PET/CT, FDG accumulation was high in the ventral portion (standardized uptake value [SUV]_max_ = 8.3), and accumulation was mild in the dorsal portion, except the border region, and comparable to background liver accumulation (SUV_max_ = 2.8; Fig. 4). No extrahepatic tumor was detected.

Based on imaging findings, the ventral portion was considered to have a malignant tumor such as cholangiocarcinoma due to the invasive nature, diffusion restriction, and high FDG uptake. The dorsal portion was suspected to comprise abundant microcystic or mucinous components and lacked invasive features. Our first imaging diagnosis was cholangiocarcinoma with abundant mucinous component, while the second diagnosis was a collision tumor of cholangiocarcinoma and schwannoma.

She subsequently underwent an extended left hepatectomy. Although inferior vena cava was compressed by the tumor, they were separable. Tumor invasion was suspected in the left and middle hepatic veins; hence, both were resected in the common trunk. The middle hepatic vein was reconstructed using the cryopreserved superior vena cava homograft. There were no intra- or post-operative complications.

**Fig. 5:**
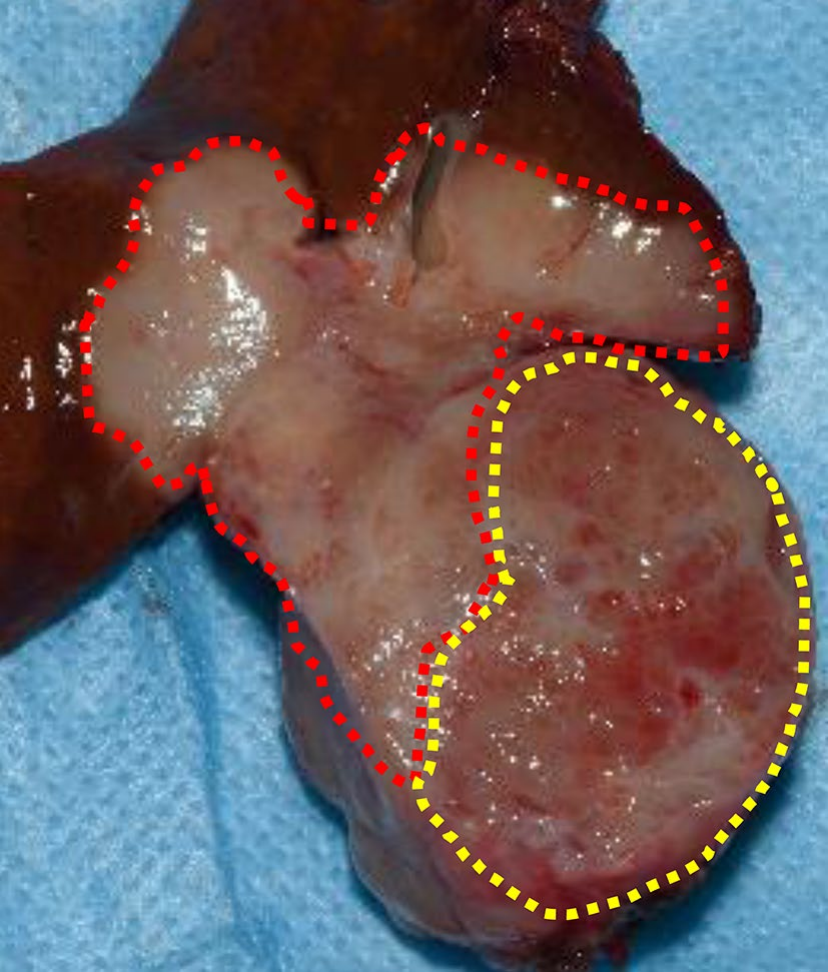
Gross pathology specimen demonstrates a mass measuring 45 × 45 × 30 mm in S1. The ventral portion is a solid mass with an irregular margin (red line). The dorsal portion exhibits multiple small cystic appearances with a well circumscribed boundary (yellow line)

**Fig. 6:**
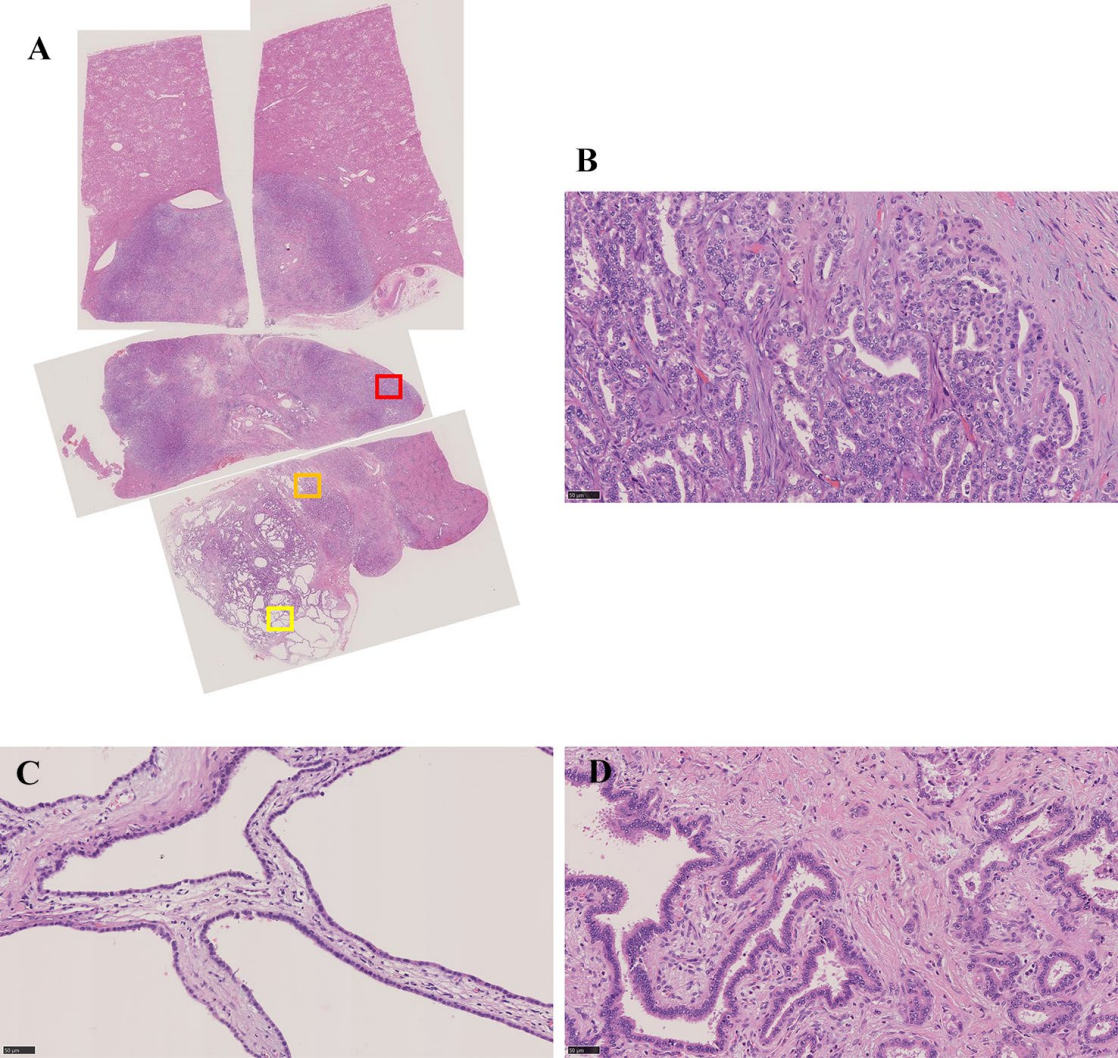
In the ventral portion, atypical cells with high nucleocytoplasmic ratios, small nucleoli, and eosinophilic cytoplasm proliferate forming a small glandular structure, consistent with a well to moderately differentiated cholangiocarcinoma (**A** H&E, loupe view, red square; **B** H&E, × 40). In the dorsal portion, bile duct-like glandular ducts proliferate with dilatations and meanders, with abundant intervening fibrous stroma. Most of the glandular ducts are composed of cuboidal or cylindrical non-mucin-secreting epithelium with little atypia, which indicates biliary adenofibroma (**A** yellow square; **C** H&E, × 40). Cholangiocarcinoma and biliary adenofibroma are intimately admixed in the border area of the dorsal and ventral portion (**A** orange square; **D** H&E, × 40)

Gross pathology specimen demonstrated a mass measuring 45 × 45 × 30 mm in S1 (Fig. 5). The ventral portion was a solid mass with an irregular margin. The dorsal portion exhibited multiple small cystic appearances with a well circumscribed boundary. The liver was not cirrhotic. The microscopic examination revealed a tumor with two different components. The ventral portion was a well to moderately differentiated cholangiocarcinoma (Fig. 6). Involvement of the middle hepatic vein was noted. In the dorsal portion, bile duct-like glandular ducts proliferated with dilatations and meanders, with abundant intervening fibrous stroma (Fig. 6). Most of the glandular ducts are composed of a cuboidal or cylindrical non-mucin-secreting epithelium with little atypia. Apocrine snouts and papillary structures were also seen. Some epithelial cells showed high grade atypia. In the border area of the dorsal and ventral portion, the two components were intimately admixed (Fig. 6). Immunohistochemically, the dorsal portion was positive for Cytokeratin 7, partially positive for Cytokeratin 19, and negative for Cytokeratin 20. The percentage of P53-positive cells was about 5% and the Ki-67 proliferation index was less than 10%. The final diagnosis was intrahepatic cholangiocarcinoma arising from biliary adenofibroma.

Currently, the patient is alive without signs of recurrence after more than 3 years of operation.

## Discussion

Biliary adenofibromas are rare, primary hepatic tumors with potential for malignant transformation. Previous literature has described tumors ranging in size from 1.8 to 16 cm, with patient ages ranging from 23 to 83 years, and no characteristic symptoms or laboratory findings [2]. Pathologically, they are characterized by microcystic and tubule-acinar glandular structures lined by non-mucin-secreting biliary epithelium and supported by a fibroblastic stromal scaffolding [1]. The suggested pre-malignant changes included papillary growths within the cysts lined by an eosinophilic epithelium with apocrine-like changes, secretory snouts, and vesicular nuclei with prominent nucleoli. Carcinoma arising from biliary adenofibroma is a conventional adenocarcinoma in most cases [10, 12, 13], as could be seen in our case. Till date, 28 cases of biliary adenofibroma have been reported in English literature [1–24]; a total of 14 cases have been associated with malignant transformation [2–13, 16, 18]. However, the frequency of malignant transformation is unknown due to the lack of large studies.

On ultrasound, two cases presented as a hypoechoic mass [2, 23], while the other four cases were mostly a hyperechoic mass [3, 4, 19, 20]. In the present case, the portion of biliary adenofibroma showed high echogenicity with a clear boundary and the portion of cholangiocarcinoma showed low echogenicity with an irregular margin. Microscopically, the hyperechogenic lesion was composed of abundant microcysts. From this finding, we hypothesized that the high echogenicity might reflect multiple ultrasound reflections from numerous tiny cysts inside the tumor like pancreatic serous cyst neoplasm [25]. Various cyst sizes composing the biliary adenofibroma might have resulted in varied echogenicities in previous reports.

On CT, biliary adenofibroma is reported as a solid-cystic mass with a well-defined margin, lobulated shape, and internal septa [2]. On MR imaging, the serous cystic component shows high signal intensity on T2-weighted images and low signal intensity on T1-weighted images, with multiple septation. Solid components and septa are likely to show low signal intensity on T2-weighted images reflecting high content of fibrous stroma. Unlike intraductal papillary neoplasm of the bile duct, communication or dilation of bile duct is not usually seen. Unlike mucinous cyst neoplasm, intracystic hemorrhage and calcifications are not common [2]. These are consistent with the present case. In addition, our case showed water-like high signal intensity on heavily T2-weighted imaging; these areas showed contrast enhancement, like pancreatic serous cystic neoplasm [25].

On enhanced CT and MR imaging, biliary adenofibroma has been reported to show a gradual delayed enhancement pattern reflecting abundant stroma [2], which is not consistent with our case showing a heterogenous early enhancement and delayed wash-out on CT. Pathologically, in the present case, the density of glandular tissue, expansion of glandular lumen, and fibrous stroma were not uniform. In some areas, tubule-acinar glandular structures were abundant, and glandular expansion and fibrous stroma were sparse; these areas may have shown early enhancement due to abundant blood flow and wash-out due to sparsity of fibrous tissue. As no atypia was seen in these areas, the pattern of enhancement might have been influenced by the proportion of each tissue rather than malignancy.

For malignant transformation, previous research has suggested the following imaging findings as specific features for malignant biliary adenofibroma and not found in benign lesions: (1) multiple lesions, (2) restricted diffusion on MR imaging, (3) obscure margin, (4) enhancement in the arterial phase followed by wash-out in the venous phase [2]. In the present case, diffusion restriction and obscure margin were observed in the malignant component and not in the benign component, which is in accordance with the previous report. However, the malignant component showed peripheral early enhancement with delayed centripetal filling, which is like conventional intrahepatic cholangiocarcinoma, and benign component showed heterogeneous early enhancement with delayed wash-out. As noted in our case, the malignant transformation generally shows conventional cholangiocarcinoma pathology; hence, the delayed centripetal enhancement may have depended on the amount of fibrous stroma of adenocarcinoma.

On FDG-PET/CT, high accumulation was seen in the malignant component and not in the benign component, thereby, clearly distinguishing between the benign and malignant component. The FDG accumulation of the malignant component was compatible with the typical findings of cholangiocarcinoma [26]. To the best of our knowledge, this is the only case showing FDG-PET/CT manifestation of biliary adenofibroma. Hence, further investigation is needed to confirm the usefulness of FDG-PET/CT in the stratification of the risk of malignant transformation.

## Conclusions

Biliary adenofibroma can be included in the differential diagnosis when the liver tumor shows a well-marginated microcystic mass with a solid component like pancreatic serous cyst neoplasm. Invasive nature, restricted diffusion, and high FDG uptake of a part of the tumor, like intrahepatic cholangiocarcinoma, might be clues of malignant transformation.
